# Prevalence and Clinical Characteristics of Probable REM Behavior Disorder in Thai Parkinson's Disease Patients

**DOI:** 10.1155/2018/7657191

**Published:** 2018-02-04

**Authors:** Patama Gomutbutra, Kittika Kanjanaratanakorn, Nantaporn Tiyapun

**Affiliations:** ^1^Division of Neurology, Department and Faculty of Medicine, Chiang Mai University, Chiang Mai, Thailand; ^2^Research Administration Section, Faculty of Medicine, Chiang Mai University, Chiang Mai, Thailand

## Abstract

**Background:**

Previous studies have shown that Parkinson's disease (PD) patients who have REM behavior disorder (PD with RBD) might be a PD subtype since they have different symptom clusters and disease trajectories from PD without RBD.

**Objective:**

To study the prevalence of PD with pRBD and to compare the clinical characteristics with PD without pRBD. The feasibility of clinical interview of items adopted from the Mayo Sleep Questionnaire was also to be determined.

**Methods:**

A total of 140 Parkinson's patients visiting neurological clinics during January to December 2016 were enrolled in this study. “Probable RBD (pRBD)” was defined as present when the patient answered “yes” to a question adapted from the first Mayo Sleep Questionnaire (MSQ). The demographic data, motor symptoms, and nonmotor symptoms were obtained.

**Results:**

The prevalence of pRBD among this study's PD patients was 48.5% (68 out of the total of 140). The median onset of RBD before PD diagnosis was 5 years (range: 0–11 years). By comparison of PD with pRBD and PD without pRBD, this study showed significant difference in the levodopa equivalent dose (742 mg/day versus 566 mg/day; *p* < 0.01), prevalence of symptomatic orthostatic hypotension (35.3% versus 8.3%; *p* < 0.01). The multivariable analysis found that pRBD is independently associated with orthostatic hypotension (OR = 5.02, *p* < 0.01). Conclusion. The findings regarding prevalence and main clinical features of PD with pRBD in this study were similar to those of a previous study of PD with polysomnogram- (PSG-) proven RBD. This study hypothesized that interviewing by adopted MSQ may be a cost-effective tool for screening RBD. Further studies with direct comparison are needed.

## 1. Introduction

### 1.1. Diagnosis and Classification of RBD

Rapid eye movement sleep behavior disorder (RBD) is parasomnia disorder characterized by clinical dream-enactment behavior ranging from vocalization to vigorous movement, resulting in sleep-related injury.

Definite diagnosis of RBD needs standard full-night polysomnography type I (monitoring devices perform in-laboratory, technician-attended, overnight polysomnography) [1] to demonstrate REM sleep without atonia (RSWA) [1] and to exclude other sleep disorders that may mimic RBD such as obstructive sleep apnea (OSA) and nocturnal epilepsy. Probable RBD (pRBD) was diagnosed clinically, and the details could be obtained from the interview [2]. There were several validated questionnaires to help clinicians gather adequate information to diagnose pRBD. Interestingly, a complex multi-item questionnaire does not show superiority upon comparison with a single question as RBD1Q [3]. However, the first core question of the Mayo Sleep Questionnaire (MSQ) [4] which was validated by PSG had acceptable sensitivity and specificity of 93%/87% and 98%/74%, respectively. MSQ also had four additional subquestions on RBD which improved specificity.

RBD is classified as follows: (1) acute RBD commonly caused by drugs or metabolism; (2) chronic RBD which is found in synucleinopathies such as Parkinson's disease (PD), dementia with Lewy bodies (DLB), and multiple system atrophy (MSA); and (3) idiopathic RBD which is now recognized as a prodromal symptom of degenerative diseases including Parkinson's disease.

### 1.2. Prevalence of RBD in Parkinson's Disease Patients

Several international reports show high prevalence of RBD in PD patients. Prevalence of definite RBD was confirmed by PSG ranging from 35% to 58% and probable RBD (pRBD), assessed by validated questionnaires, ranging from 15% to 45% [5]. Two concurrent studies in Bangkok using composite nonmotor questionnaire showed that 40–48% of PD had vivid dreams or dream enactment activity [6, 7]. However, prevalence by PSG-proven RBD has not yet been explored in Thailand.

### 1.3. PD with RBD as a PD Subtype?

Reports regarding clinical characteristics and pathology distinction between PD with RBD and PD without RBD were varied. Most studies reported that RBD in PD was associated with more akinetic rigid symptoms and nonmotor symptoms such as orthostatic hypotension, cognitive impairment, and depression. These associations were observed even after adjusting potential confounders such as age, duration of disease, clonazepam usage, and dosage of levodopa medication [8].

Despite an increase in the number of publications on the clinical features of PD with RBD in the past 10 years, studies that focus on the clinical characteristics of RBD in Thai PD patients are lacking [9].

### 1.4. Objective of Study

To study the prevalence of probable RBD in patients with Parkinson's disease (PD with pRBD) by clinical interviews and to compare the clinical characteristics with PD without probable RBD (PD without pRBD), usefulness of clinical interviews using items adapted from the Mayo Sleep Questionnaire (MSQ) was also determined.

## 2. Materials and Methods

### 2.1. Study Population

The 140 subjects were chosen from the neurology clinics at Maharaj Nakorn Chiang Mai Hospital during the period from January 1, 2016, to December 31, 2016. Subject enrollment inclusion's criteria included idiopathic Parkinson's disease diagnosed by a neurologist using the UK Parkinson's Disease Society Brain Bank Diagnostic Criteria. The patients had been followed up by the clinic for at least 6 months until the time of enrollment. Patients who could not communicate in Thai language did not have a care giver who could provide clinical information and had possible OSA diagnosed by asking a screening question were excluded. The Ethics Committee of Chiang Mai Medical University approved the study, and the participants' voluntary written informed consent was obtained (REC-25590427-06956).

### 2.2. Measurement

#### 2.2.1. Baseline Clinical Features of PD

The demographic data including age, gender, disease duration, Hoehn and Yahr stage, and dosage of levodopa were obtained from the medical records. Levodopa equivalent doses were estimated using a web-based calculator: http://www.parkinsonsmeasurement.org/toolBox/levodopaEquivalentDose.htm [10].

#### 2.2.2. Motor Symptoms

The cross-sectional symptoms of tremor, rigidity, and freezing of gait were evaluated by neurologists in the researchers' team with the checklist provided (present/absent). The motor fluctuations defined either dyskinesia or predictable, or both, and details regarding the same were obtained from the patient's diary, recorded 3 days before visiting.

#### 2.2.3. Nonmotor Symptoms

Diagnosis of dementia is defined by the previous diagnosis by a neurologist and MMSE < 26 [11] or less. Depression, apathy, and hallucination in this study refer to those who were diagnosed through psychiatric consultations.

Symptomatic orthostatic hypotension [12] is defined as a patient having had the symptom plus evidence supporting neurogenic orthostatic hypotension (BP when standing upright was lower than that in the seated position by at least 20/10, and HR at 3 min increases by <15/min). The screening question was as follows: “In the past 3 months, have you commonly experienced any of the following symptoms when you stand up or within 3–5 min of standing up, which gets better when you sit or lay down: feeling faint, dizziness, visual disturbances, difficulty in breathing, and leg buckling?” Patients who responded positively to the screening questionnaire were asked to proceed for evaluation of sitting and standing blood pressures. The blood pressure was measured by one of the research team members, using a digital blood pressure monitor (Omron (C) 7130). The patients were told to sit down, and the BP and HR were measured after at least 5 min of sitting; then, the patients were told to stand up, and the measuring of BP and HR was repeated after standing for 3 min [12].

#### 2.2.4. Screening for Probable RBD

Patients who volunteered to participate in the research were interviewed by the study nurse with a question which was adapted from the first question of the Mayo Sleep Questionnaire by asking in Thai or northern Thai language, “Have you ever seen the patient appearing as if “acting out his/her dreams” (punched or flailed arms in the air, shouted, or screamed) while sleeping?” The patients who answered yes were defined as PD with pRBD, and the details of RBD were explored.

#### 2.2.5. Clinical Characteristics of PD with pRBD

The PD with pRBD patients were interviewed in detail regarding the following:

1. Acting out the dream, with modified Derry's frontal lobe epilepsy parasomnia scale [13] which included age at the onset, duration, clustering, timing, symptoms, stereotypy, recall, and vocalization
2. Medication in the past 3 months
3. Dream content

The open task to PD with positive RBD screening was to “narrate their dream as much as they could.” If they had variety of dream content, they were asked, “Which was the most memorable one?”

Later, the dream content was grouped into five categories: (1) chase or defense against attack by person; (2) chase or defense against attack by animal; (3) accident involving self, for example, drowning or falling from cliff; (4) nonviolent dream, for example, sport, work, or daily activity; and (5) cannot remember dream content.

### 2.3. Statistical Analysis

#### 2.3.1. Sample Size Calculation

The expected prevalence was between 10% and 50%; therefore, the minimum sample size was calculated using *p*=0.10, and the maximum sample size was calculated using *p*=0.50; the significance level = 0.05, and the precision (*d*) of *p*=0.05 [14]:

(1) $$\begin{array}{c} n=\frac{Z\cdot P\left(-P\right)}{d^{2}}, \end{array}$$

where *n* = the sample size and *Z* = *Z* statistic: for the level of confidence of 95%, which is conventional, the *Z* value is 1.96. The sample size would be a minimum of 138 and a maximum of 384.

#### 2.3.2. Data Analysis

The basic demographic data, including percentage, average, standard deviation, median, and range, were analyzed by descriptive statistics. Student's *t*-test was used for analyzing quantitative data; categorical data were compared between groups using the chi-square test or Fisher's exact test. All statistical tests were performed at 5% significance level.

## 3. Results

### 3.1. General Data regarding Sample Population

A total of 140 patients were included in this study, and their average age was 65.3 years, with males comprising 51%. Most of the information (70%) was obtained from their bed partners. The prevalence of pRBD in this study was 48.5% ± SE 4.2%.

Upon comparison of PD with pRBD and PD without pRBD, this study showed significant difference in the levodopa equivalent dose (742 mg/day versus 566 mg/day; *p* < 0.01), prevalence of symptomatic orthostatic hypotension (35.3% versus 8.3%; *p* < 0.01), and motor fluctuation (48.5% versus 31.9%; *p*=0.04), while there were no significant differences in disease duration, tremor, and severity of disease by the Hoehn and Yahr stage. The details of clinical characteristics of comparison between PD with pRBD and PD without pRBD are demonstrated in Table 1.

### 3.2. Characteristics of RBD in Patients with pRBD Screened Using Mayo Sleep Questionnaire

The median onset of RBD before PD diagnosis was 5 years (range 0–11 years). The proportion of noninjurious to injurious RBD was 80  :  20. The details of characteristics of comparison between PD with pRBD and PD without pRBD are demonstrated in Table 2.

### 3.3. Analysis of the Association of LED, pRBD, H&Y Stage, Hypotension, and Motor Fluctuation

Since high LED was described in previous studies as a major factor contributing orthostatic hypotension and motor fluctuation [6, 7, 15], we perform an additional analysis to illustrate whether the higher percentage of orthostatic hypotension and motor complication were caused by levodopa dosage or RBD.  Table 3 divided the patients into different groups of whether high LED (defined by the median dosage of our population was about 600 mg/day) showed that pRBD, advance H&Y stage, orthostatic hypotension, and motor fluctuation had significantly higher proportion fall in high LED group.

We performed the multivariate logistic regression analysis for factors associated with orthostatic hypotension and motor fluctuation. We select factors to include in the multivariated model from the bivariate corelational analysis. The three factors pRBD, LED, and H&Y stage were included into the orthostatic hypotension model as Table 4. The factors significantly associated with orthostatic hypotension were pRBD (OR 5.02, *p* < 0.01) and LED (OR 1.003, *p* < 0.01). The two factors pRBD and LED stage were included into motor fluctuation model as Table 5, only LED (OR 1.004, *p* < 0.01) while pRBD failed to have independent association (OR 1.57, *p*=0.22).

## 4. Discussion

### 4.1. Prevalence and Demographic Data of pRBD

The prevalence of pRBD in the PD subjects of this study was 48.5%; among this, 30% was newly diagnosed through the interview using the adapted Mayo Clinic Sleep Questionnaire. This finding was consistent with a previous report in Thai PD that sleep problems were often underdiagnosed (59.2% detected by physicians, compared to 81.7% through MPDSS questionnaire) [16]. The average age of onset of RBD in this study was 50, and the patients were predominantly male (56%). The symptoms of RBD preceded the onset of motor symptoms of PD in the median of 5 years. The results of this study were similar to several previous reports [4, 8, 17].

### 4.2. Comparison of Clinical Characteristics between PD with pRBD and PD without pRBD

Remarkable higher levodopa equivalent dose (LED), symptomatic orthostatic hypotension, and motor fluctuations were observed in patients having PD with pRBD by comparison with those having PD without pRBD. These findings in this study's PD with pRBD are similar to the findings of a previous study that compared PD with PSG-proven RBD [8].

The multivariate analysis to explore relationship between pRBD, LED, and orthostatic hypotension found that pRBD is an independent factor associated with symptomatic orthostatic hypotension after adjust for age, H&Y, and daily dose levodopa.

Levodopa-related blood pressure declination has been reported to be in the ranges of 4.6–20 mmHg in systolic blood pressure and 2.1–5.0 mmHg in diastolic blood pressure [15]. However, symptomatic orthostatic hypotension in Parkinson's disease is more likely to be due to an interaction between autonomic failure because of the disease itself and a combination of dopaminergic medications and other medications causing low SBP and suboptimal hydration. RBD may also be related to more severe autonomic dysfunction as evidenced from the study uptake of ^123^I-labeled metaiodobenzylguanidine (MIBG) in myocardial scintigrams, showing that RBD is an independent factor associated with low uptake indicating reduced cardiac sympathetic ganglia function [18]. Meanwhile the association of pRBD with motor fluctuation is confounded by the higher LED and H&Y stage. These findings are consistent with a recent systematic review. Orthostatic hypotension was consistently associated with RBD. Meanwhile motor fluctuation was not clearly associate with RBD [19].

The findings which were different from the findings of the previous studies in PD with proven RBD are as follows: there was no significant difference in terms of predominant tremor or akinetic rigid syndrome. The explanation for this might be that because of the cross-sectional nature, the patient's symptoms might have disappeared after treatment or during more advanced disease stages. In addition, this study did not show any statistical difference in cognitive dysfunction between the two groups.

Our study cannot conclude whether pRBD associates with more advanced diseases since there was no statistically significant difference in the duration of the disease and the proportion of patients with advanced H&Y stage between the two groups. However, we are aware of the higher proportion of advanced H&Y stage in PD with pRBD (41.2% versus 34.7%) and its gross measurement in nature. Using the finer scale as UKPDS may help us to clarify this issue.

### 4.3. Acting Out Dream Behavior and Dream Content in RBD

Most (80%) of the dream enactment behaviors were classified as the noninjurious type. The most common type was “purely vocalization.” The most consistent RBD characteristic among this study's PD with pRBD was “late night” onset; however, not many (17%) of this study's patients or their partners reported behavior related to “dream content.”

Additionally, this study showed that “violent dreams” was the most common (60%) and memorable one among PD with RBD patients. This result is close to the finding of a PSG study in Thai PD [20].

### 4.4. Restrictions and Recommendations

It was realized that, in this study, the limitations of RBD diagnosis were not confirmed by PSG and recall bias of acting out behavior. However, this may also indicate that evaluating RBD in Parkinson's disease patients with more specific questions about dream content may be helpful in formulating tailor-made therapeutic options.

Since the standard full-night polysomnography type I is expensive, labor intensive, and time consuming, it is not practical to use it in routine practice for diagnosis in all PD patients. A highly sensitive and specific tool such as PSG is reasonably good for idiopathic RBD and to exclude other sleep or epileptic disorders; however, this issue is different from simple RBD in PD. A simpler tool with moderate sensitivity such as a questionnaire (it should be validated once with PSG) might be more realistic for PD. Therefore, the researchers agree with some experts' opinion that “outside of research settings, it may be reasonable to diagnose RBD in PD empirically and to investigate further only if treatment response or clinical presentation is atypical [21].”

## 5. Conclusion

In this study, prevalence of PD with pRBD was observed in nearly half of the PD patients, which is a finding close to that of previous reports. The main clinical features of pRBD in this study were similar to those of previous studies on PD with PSG-proven RBD: occurrence at late night and dream content related to violence. PD with RBD in this study was also associated with high levodopa dosage, symptomatic orthostatic hypotension, and motor fluctuations. Therefore, this study supported that the adapted Mayo Sleep Questionnaire Screening for pRBD in PD might be cost effective to diagnose pRBD in comparison with the high-cost and labor-intensive standard full-night PSG. All the same, further studies are needed with direct comparison questionnaires and PSGs in the same cohort. In addition, it needs to be mentioned that RBD in PD might reflect high disease severity, which calls for special medical attention regarding prescription and monitoring.

## Figures and Tables

**Table 1:** Comparison of characteristics between PD without RBD and PD with RBD.

Variables	PD without pRBD (*N* = 72)	PD with pRBD (*N* = 68)	*p* value
Age (year: mean ± SD)	65.0 ± 10.2	63.1 ± 9.7	0.54
Gender: male: *n* (%)	35 (48.6%)	38 (55.9%)	0.39
Disease duration (year: mean, range)	4.9 ± 2.9	5.0 ± 3.4	0.96
Hoehn and Yahr stage: stages 3, 4: *n* (%)	25 (34.7%)	28 (41.2%)	0.43
Levodopa equivalent dosage (mg/day: mean ± SD)	566 ± 325	742 ± 527	**<0.01**
*Motor symptom*			
Tremor: *n* (%)	50 (69.4%)	55 (80.9%)	0.12
Rigidity: *n* (%)	48 (66.7%)	52 (76.5%)	0.20
Freezing of gait: *n* (%)	18 (25.0%)	25 (36.8%)	0.13
Motor fluctuation symptom: *n* (%)	23 (31.9%)	33 (48.5%)	**0.04**
*Nonmotor symptom*			
*Symptomatic orthostatic hypotension*: *n (%)*	6 (8.3%)	24 (35.3%)	**<0.01**
Morning dizziness without OH: *n* (%)	8 (11.1%)	15 (22.1%)	0.08
Depression or apathy: *n* (%)	13 (18.1%)	15 (22.1%)	0.55
Hallucination: *n* (%)	12 (16.7%)	9 (13.2%)	0.58
Diagnosed dementia: *n* (%)	14 (19.4%)	12 (17.6%)	0.45

**Table 2:** Characteristics of RBD.

Variable	(*N* = 68)
Male gender: *n* (%)	38 (55.9%)
Age of RBD onset (year: mean ± SD)	55.9 ± 8.94
Duration from RBD onset to PD diagnosis (year: median, range)	5 (0–15)
RBD notice after PD was diagnosed: *n* (%)	10 (14.7%)
*Dream enactment behavior*	
Noninjurious RBD	
Vocalization only: *n* (%)	39 (57.3%)
Vocalization with noninjurious limb movement: *n* (%)	17 (25.0%)
Injurious RBD	
Falling out of bed: *n* (%)	8 (11.7%)
Injury to bed partner: *n* (%)	4 (5.8%)
*The most rememberable dream content*	
Human- or ghost-related violence: *n* (%)	35 (51.5%)
Animal-related violence: *n* (%)	8 (11.8%)
Accident involving self, such as drowning, falling: *n* (%)	4 (5.9%)
Nonviolent acts, such as working, traveling: *n* (%)	9 (13.2%)
Cannot remember: *n* (%)	12 (17.6%)
*Details of phenomenon*	
Duration of each episode (2–10 min): *n* (%)	47 (69.1%)
Times per night (once per night): *n* (%)	58 (85.3%)
Period of night (late night): *n* (%)	63 (92.6%)
Dystonic feature (number): *n* (%)	52 (76.5%)
Stereotype (number): *n* (%)	43 (63.3%)
Recall (vividly): *n* (%)	46 (67.6%)
Behavior related to dream content: *n* (%)	12 (17%)
*Medication*	
Clonazepam: *n* (%)	45 (69.2%)
SSRI: *n* (%)	2 (0.03%)
Melatonin: *n* (%)	8 (11.7%)
Dopamine agonist: *n* (%)	16 (23.5%)
Cholinesterase inhibitor: *n* (%)	5 (7.3%)

**Table 3:** Comparison of characteristics between PD with low and high LED.

Variables	Levodopa equivalent dose (LED)		*p* value
	Less than 600 mg/day	600 mg/day or more	
pRBD: *n* (%)	23 (33.9%)	45 (66.1%)	<0.01
Hoehn and Yahr stage: stages 3 and 4: *n* (%)	14 (24.5%)	39 (73.6%)	<0.01
Orthostatic hypotension: *n* (%)	6 (20.0%)	24 (80.0%)	<0.01
Motor fluctuation: *n* (%)	19 (33.9%)	37 (66.1%)	0.03

**Table 4:** The multivariable analysis of factors associated with orthostatic hypotension.

Factors	Odds ratio	95% confidence interval		
		Lower	Upper	*p* value
pRBD (yes)	5.018	1.731	14.545	<0.01
LED (mg/day)	1.003	1.001	1.004	<0.01
H&Y stage 3 or 4 (yes)	1.143	0.406	3.219	0.80

**Table 5:** The multivariable analysis of factors associated with motor fluctuation.

	Odds ratio	95% confidence interval		*p* value
		Lower	Upper	
pRBD	1.576	0.763	3.256	0.22
LED	1.002	1.001	1.003	<0.01
